# Alcohol? Not for Non-alcoholic Fatty Liver Disease Patients

**DOI:** 10.3389/fmed.2020.00014

**Published:** 2020-02-12

**Authors:** Francesca Cainelli, Titya Thao, Carolyne Pung, Sandro Vento

**Affiliations:** ^1^Raffles Medical Group Clinic, Phnom Penh, Cambodia; ^2^Faculty of Medicine, University of Puthisastra, Phnom Penh, Cambodia

**Keywords:** non-alcoholic fatty liver disease, non-alcoholic steatohepatitis, liver fibrosis, alcohol, liver cirrhosis, hepatocellular carcinoma

## Introduction

Non-alcoholic fatty liver disease (NAFLD) is one of the most common causes of chronic liver disease worldwide, with an estimated global prevalence of 25%, liver-specific mortality of 0.77 persons per 1,000, and overall mortality of 11.77 per 1,000 person-years (1). It has long been debated whether alcohol consumption influences the course of NAFLD. Three of the most recent studies have shed light over this important issue.

In a study conducted in the United States, alcohol consumption of at least 30 g/day for men and 15 g/day for women was associated with increased mortality in individuals with fatty liver and metabolic syndrome (2). In a Finnish study, even low alcohol intake in fatty liver disease was associated with increased risks for advanced liver disease and cancer (3). Finally, in a South Korean study, even though low alcohol consumption was linked to a decreased risk of incident hepatic steatosis, the same levels of low alcohol consumption were associated with a higher risk of developing advanced liver fibrosis in those subjects who progressed to more severe NAFLD over time (4).

## Recent Studies

In the first study (2), the authors searched the National Health and Nutrition and Examination Survey III for 20–74 year old individuals with liver steatosis (detected by ultrasound), for whom mortality and follow-up data were available, gathered data from the self-reported alcohol use questionnaire, and calculated the average amount of alcohol consumption in drinks/day for each participant during the year preceding enrollment. The study cohort included 4,264 individuals with hepatic steatosis of whom 46% had metabolic syndrome (2). Overall mortality was significantly higher in subjects with excessive alcohol consumption (>3 drinks/day for men and >1.5 drinks/day for women) after both 5 years and mean 20 years of follow-up. In multivariate analysis, the presence of metabolic syndrome and of excessive alcohol consumption were independently associated with an increased risk of death in the presence of hepatic steatosis, and in a subgroup analysis, the association of excessive alcohol use with cancer and cardiovascular mortality was significant in individuals with metabolic syndrome (2). The study had its limitations (self-reported use of alcohol, one-time collection, use of ultrasound findings rather than liver histology to determine fatty liver), but the population-based huge cohort reflecting real-life practice is certainly an important strength.

The Finnish study included 8,345 subjects with hepatic steatosis participating in the health-examination surveys FINRISK 1992–2012 or Health 2000 and with available data on baseline alcohol intake (3). The authors linked the data with national registers for hospital admissions, cancers, and death from liver, cardiovascular, and malignant diseases, and all-cause death, and adjusted for various confounders (3). The diagnosis of fatty liver disease was not based on ultrasound but rather on a fatty liver index ≥60.5. Alcohol use was associated with a dose-dependent risk increase for incident advanced liver disease and cancers. Consuming just 10–19 g/day of alcohol, or only 0–9 g/day as non-wine beverages, doubled the risk for advanced liver disease. Alcohol intake above 30 g/day increased mortality risk compared to lifetime abstainers (3). In contrast, alcohol intake up to 49 g/day showed a 22–40% reduction of incident cardiovascular disease, but only in never smokers. The main limitation of this robust study is that alcohol habits were assessed only once, even though alcohol use over time was analyzed in a subpopulation and found to be largely stable.

The South Korean study involved an impressive cohort of 190,048 young and middle-aged men and women without NAFLD at baseline and with low alcohol consumption (within the limits allowing a diagnosis of NAFLD) with a maximum of almost 16 years of follow-up (4). The modest alcohol consumption was linked to a decreased risk of incident hepatic steatosis but in the small subgroup of subjects (around 6%) who progressed to more severe NAFLD over time; the same low levels of alcohol consumption were associated with a higher risk of developing hepatic steatosis and with intermediate/high chances of advanced liver fibrosis (4). Interestingly, the association between modest drinking and the development of incident liver steatosis and an intermediate/high fibrosis score was observed in both obese and non-obese individuals (4). This impressive study has its main limitation in the fact that the diagnoses of liver steatosis and fibrosis were not based on liver biopsy, but rather on ultrasound (for steatosis) and on two non-invasive (although validated) hepatic fibrosis scores.

It should be noted that these studies have been conducted in a largely Caucasian North American population (2), in Finnish (3), or in Northeast Asians (4), and should be replicated in other racial groups.

## Discussion

How can the results of these three studies be put in context? Liver inflammation can also be influenced by even modest alcohol consumption, as in one United States-paired biopsy longitudinal study examining NAFLD progression; patients who drank ≤ 2 drinks daily had less improvement in steatosis and lower probability of non-alcoholic steatohepatitis resolution over average 4 years of follow-up, when compared to patients who did not drink at all (5). A Japanese longitudinal study of 301 patients with histopathologically diagnosed NAFLD showed that over average 6 years of observation, those who drank modestly (<20 g alcohol daily), especially if they had advanced fibrosis, developed hepatocellular carcinoma significantly more often than non-drinkers (6).

Can the alcohol-related damage to the liver be somehow balanced by a beneficial effect on the cardiovascular system, taking into account that cardiovascular disease (CVD) is considered the leading cause of death among patients with NAFLD (7)? In the Coronary Artery Risk Development in Young Adults longitudinal cohort study (a population-based, prospective study of 5,115 black and white young adults, 18–30 years old, followed for 25 years in the United States), 570 participants had NAFLD at a mean age of 50 years, and 58% were modest drinkers. No significant differences were found between drinkers and abstainers in CVD risk factors (hypertension, diabetes, hyperlipidemia) or subclinical CVD measures [such as coronary artery calcification, early transmitral velocity/late (atrial) transmitral velocity ratio] (8).

In conclusion, considering that NAFLD patients may have advanced fibrosis, that alcohol increases the risk of cancer at all level of consumption (9), that patients can underreport their drink consumption by 50–66% (10), and that it is difficult for them to strictly adhere to advice related to alcohol use, patients with non-alcoholic fatty liver disease should be strongly advised to completely abstain from alcohol.

## Author Contributions

FC had the idea of writing the manuscript and drafted it. TT and CP co-drafted the manuscript. SV contributed to the drafting, corrected, and reviewed the manuscript. All the authors approved the final version.

### Conflict of Interest

The authors declare that the research was conducted in the absence of any commercial or financial relationships that could be construed as a potential conflict of interest.
